# A U.S. survey of pre-operative carbohydrate-containing beverage use in colorectal enhanced recovery after surgery (ERAS) programs

**DOI:** 10.1186/s13741-021-00187-3

**Published:** 2021-05-28

**Authors:** Sunitha M. Singh, Asha Liverpool, Jamie L. Romeiser, Joshua D. Miller, Julie Thacker, Tong J. Gan, Elliott Bennett-Guerrero

**Affiliations:** 1 Department of Anesthesiology, Stony Brook University Medical Center, 101 Nicholls Road, Health Science Center, L-4, 060, Stony Brook, NY 11794-8480 USA; 2 Department of Medicine/Endocrinology, Stony Brook University Medical Center, 101 Nicolls Road, Health Science Center, L-4, 060, Stony Brook, NY 11794-8480 USA; 3 Department of Surgery, Duke University Medical Center, 10 Duke Medicine Circle, Durham, NC 27710-1000 USA

**Keywords:** Enhanced recovery, ERAS, Pre-operative, Carbohydrate-containing beverage, Insulin, Diabetes

## Abstract

**Background:**

Carbohydrate-containing drinks (CCD) are administered preoperatively in most enhanced recovery after surgery (ERAS) programs. It is not known which types of CCDs are used, e.g., simple vs. complex carbohydrate, and if the choice of drink differs in patients with diabetes.

**Methods:**

A national survey was performed to characterize the use of preoperative CCDs within the context of adult colorectal ERAS programs. The survey had questions regarding the use of preoperative CCDs, the types of beverages used, and the timing of beverage administration. The survey was administered electronically to members of the American Society for Enhanced Recovery (ASER) and manually to participants at the 2018 Perioperative Quality and Enhanced Recovery Conference in San Francisco, CA.

**Results:**

Responses were received from 78 unique hospitals with a colorectal ERAS program of which 68 (87.2%) reported administering a preoperative drink. Of these, 98.5%, 80.9%, and 60.3% of hospitals administered a beverage to patients without diabetes, patients with diabetes not taking insulin, and patients with diabetes taking insulin, respectively. Surprisingly, one third of programs that administered a beverage to patients with diabetes used a simple carbohydrate drink.

**Conclusions:**

This survey finds a high use of CHO-containing beverages in colorectal ERAS programs. More than half of all programs administer a CHO-containing beverage to patients with diabetes, and surprisingly, there is significant use of simple carbohydrate beverages in patients with diabetes receiving insulin.

**Supplementary Information:**

The online version contains supplementary material available at 10.1186/s13741-021-00187-3.

## Background

Enhanced recovery after surgery (ERAS) programs are evidence-based, multidisciplinary, surgical care pathways, which have become widespread over the past 2 decades (Pogatschnik & Steiger, 2015; Ackerman et al., 2020). ERAS programs were initially implemented in elective colorectal surgeries, and their use within this service line has been well studied (Carey & Hogan, 2021; Burden et al., 2012). This research has contributed to expansion of ERAS programs to additional surgical service lines (e.g., thoracic, cardiac, gynecology and orthopedic surgeries) (Ackerman et al., 2020; Desiderio et al., 2018). The effectiveness of ERAS programs, comprised of approximately 20 elements, is likely due to its ability to standardize care for patients undergoing elective surgery (Carey & Hogan, 2021; Desiderio et al., 2018; Gramlich et al., 2017). Despite the now ubiquitous nature of ERAS programs, research regarding individual elements is limited.

In most ERAS programs, preoperative carbohydrate-containing drinks (CCD) are a common component. CCDs have been shown to improve the endocrine and metabolic response to surgical stress as well as reduce pre-operative discomfort, e.g., thirst, hunger, anxiety (Pogatschnik & Steiger, 2015; Ackerman et al., 2020; Hausel et al., 2001; Gustafsson et al., 2008; Talutis et al., 2020; Tall & Nygren, 2020; Loodin & Hommel, 2020; Çakar et al., 2017). Preoperative CCDs include simple carbohydrates, e.g., apple juice, or complex carbohydrates, e.g., maltodextrin, for which there are multiple commercial preparations. Little is known about which preoperative CCDs are more frequently used in ERAS programs across United States (U.S.) hospitals. This is particularly relevant to patients with diabetes, where the use of preoperative CCDs might impact perioperative glycaemia, potentially leading to delay or case cancelation (Cua et al., 2021). Moreover, if surgery occurs, the dysglycemia could be detrimental (American Society of Anesthesiologists Committee, 2011). Alternatively, it would be unfortunate if CCDs are withheld from patients with diabetes in order to avoid hyperglycemia when in fact their use is proven safe and effective, since allowing patients with diabetes to avoid preoperative fasting may be particularly beneficial (Ackerman et al., 2020; Gustafsson et al., 2008; Talutis et al., 2020; Tall & Nygren, 2020).

Therefore, we conducted a survey to characterize the use and type of pre-op CCDs drinks in adult colorectal ERAS programs across the U.S. By identifying how ERAS pathways are using pre-operative drinks, we aim to inform the ERAS community on current practices regarding the use of preoperative CCDs.

## Methods

### Data collection

Institutional review board (IRB) approval was obtained from the Stony Brook University IRB. The brief 9-question survey, available as Additional file 1, asked questions regarding the use of preoperative CCDs, including the types of beverages used, the timing of beverage administration, and institutional demographics. The survey underwent a multidisciplinary review during the design and test phase and was programmed with branching logic to help minimize “missing data.” The survey was circulated electronically using the Qualtrics® (Provo, UT) online software to members of the American Society for Enhanced Recovery between September and November 2018. The survey was also administered to participants at the October 2018 Perioperative Quality and Enhanced Recovery Conference in San Francisco, CA.

### Statistical analysis

Analyses were performed at the institutional level. To not over represent, i.e., double-count, institutions in the study, only one response per institution was chosen. The first, most completed survey response submitted was used. Duplicate responses from institutions, surveys with unknown institutions, and non-U.S. site responses were excluded from the analysis. Responses that were missing overall beverage data (i.e., respondents did not indicate if they provided beverages by patient type) were excluded from that patient type analysis. Additionally, some responses that were missing or listed multiple drinks in the “other” category were unable to be assigned a single type of carbohydrate category (i.e., simple or complex) and were therefore excluded from the type of carbohydrate by patient type analyses. Differences in dependent proportion tests were used to compare beverage use and beverage carbohydrate (CHO) types between patients with diabetes taking insulin and patients without diabetes (Wild & Seber, 1993).

## Results

Approximately 292 surveys were distributed (~ 192 electronically and 100 in-person), and 148 completed surveys were received; thus, the estimated response rate was 50.7%. The completed surveys represented 88 unique U.S. hospitals, 78 (88.6%) of which had colorectal ERAS programs. Sensitivity analyses confirmed that responses where similar for electronically and manually administered surveys. Respondents identified as anesthesiologists (44.3%), ERAS coordinator (19.3%), surgeon (14.8%), nurse (10.2%), certified registered nurse anesthetist (3.4%), other (3.4%), nurse practitioner (2.3%), and dietitian (2.3%).

Of the 78 adult colorectal ERAS programs, 68 (87.2%) hospitals reported that they administer a CCD prior to colorectal surgery. Of these 68 colorectal programs using a preoperative CCD, 98.5% administered a beverage to patients without diabetes, 79.7% administered a beverage to patients with diabetes not taking insulin, and 60.9% administered a beverage to patients with diabetes taking insulin (*p* < 0.05). Table 1 shows use of CCDs in these subgroups. The most common simple sugar CCDs used in all populations was Gatorade®, followed by apple juice. The most common complex sugar CCDs used in all populations were ClearFast® and Ensure Pre-surgery®; see Table 2. Ninety-seven percent of programs reported administration of the CCD on the morning of surgery, and half of these hospitals also gave it the night before.

**Table 1:** Preoperative CCD use in colorectal ERAS programs

Colorectal ERAS programs using a preoperative CCD (***n*** = 68)	Given to patients without diabetes		Given to patients with diabetes not receiving insulin		Given to patients with diabetes receiving insulin		Difference in dependent proportions test
	***n*** (%)	95% CI	***n*** (%)	95% CI	***n*** (%)	95% CI	
**Use of any beverage**	**67 (98.5)**	**92.1–100**	**55 (80.9)**	**69.8–89.4**	**42 (60.3)**	**47.7–72**	***p*** **< 0.05**
**Type of carbohydrate**							
**Simple**	**23 (37.7)**	**25.5–49.9**	**17 (33.3)**	**20.8–47.9**	**13 (34.2)**	**19.6–51.4**	***P*** **= ns**
**Complex**	**38 (62.3)**	**49–74.4**	**34 (66.7)**	**52.1–79.2**	**25 (65.8)**	**48.7–80.4**	***P*** **= ns**

**Table 2:** Specific preoperative CCD use reported in colorectal ERAS programs

Colorectal ERAS programs using a pre-op CCD (***n*** = 68)	Given to patients without diabetes	Given to patients with diabetes not receiving insulin	Given to patients with diabetes receiving insulin
	***n*** (%)	***n*** (%)	***n*** (%)
**Clear-Fast®**	**18 (29.5)**	**16 (31.4)**	**12 (31.6)**
**Ensure Pre-Surgery**®	**17 (27.9)**	**16 (31.4)**	**11 (29)**
**Gatorade**®	**16 (26.2)**	**11 (21.6)**	**10 (26.3)**
**Apple juice**	**6 (9.8)**	**5 (9.8)**	**2 (5.3)**
**Other**	**4 (6.6)**^**1**^	**3 (5.9)**^**2**^	**3 (7.9)**^**3**^

^1^Patients without diabetes—other (4) includes 1 Gatorade® zero, 2 Glycemic Endothelial Drink™ (G.E.D.), and 1 Ensure® clear

^2^Patients with diabetes not receiving insulin—other (3) includes 1 Gatorade® zero and 2 G.E.D.™

^3^Patients with diabetes receiving insulin—other (3) includes 1 Gatorade® zero, 1 Gatorade® prime, and 1 G.E.D.™

Surprisingly, 34% of the institutions that administered CCDs to patients who take insulin to manage their diabetes used a simple CHO beverage. As shown in Table 1 across these patient subgroups, the proportion of simple CHO and complex CHO use was evenly distributed (within 5 percentage points). The type of CCD used was not statistically different between patients without diabetes vs. patients with diabetes taking insulin (*p* = NS). The majority of respondents (84%) reported using the same preoperative CCD beverage in other, i.e., non-colorectal, ERAS pathways at their hospital.

## Discussion

Studies have shown that traditional preoperative fasting places patients in a catabolic state, thereby increasing their risk of adverse effects related to the surgery. Preoperative CCD use mitigates this risk by maintaining patients in an anabolic state, allowing for better glycemic control and muscle preservation (Pogatschnik & Steiger, 2015; Ackerman et al., 2020; Talutis et al., 2020; Tall & Nygren, 2020). Neither the ERAS Society USA, the American Society for Enhanced Recovery (ASER), or the American Diabetes Association have promulgated specific guidelines on the use of preoperative CCDs. In 2013, Gustaffson et al. reviewed the current evidence and strongly recommended the routine use of preoperative CCDs but only weakly recommended administering these beverages to patients with diabetes (Gustafsson et al., 2012). Other than the suggestion that fluids contain preferably at least 45 g of complex CHO, there is no clear consensus on which CCDs to use (Ackerman et al., 2020; Thiele et al., 2016). Thus, the use of preoperative CCDs among U.S. ERAS programs and among patients with diabetes is unclear.

We were surprised to find that the ERAS programs we surveyed did not appear to vary in the types of beverage they provided to patients with or without diabetes. This is different from the practice at our center (Stony Brook), where, based on the theoretical risk of perioperative hyperglycemia, we do not administer a CCD (simple or complex) to patients with known diabetes.

Our study has several potential limitations. The goal of this survey was not to assess the impact of CCDs on perioperative glycemia or any other outcomes, e.g., post-operative nausea and vomiting; therefore, we cannot comment on what is the best practice in this regard. Additional study is needed on the potential impact of administration of CCDs to patients with diabetes prior to surgery. The survey underwent a multidisciplinary review prior to distribution. Though the survey was administered electronically and manually, possibly leading to inconsistencies, we found that the results were similar for electronic and manual survey responses. This is not surprising as we would not expect these results to differ substantially based on how the survey was delivered. While multiple fail safes were implemented (e.g., branching logic) to minimize missing data or multiple answers, we did have a few responses that needed to be excluded from analyses. Overall missing data was rare (only 2/67 surveys did not specify a beverage type for any of the patients with diabetes/without diabetes groups). However, a handful of respondents who specified “other” as the beverage type did not further explain the type or listed multiple types of beverages that were both simple and complex. This led to the exclusion of this data for the carbohydrate analyses. Finally, similar to most surveys, we asked respondents to report on when the CCD was administered instead of prospectively obtaining source documents/logs showing exact dates/times of beverage administration. Therefore, it is possible that their responses do not accurately reflect what really happens to patients at their site.

Whether or not the timing and frequency of administration of CHO-containing beverages matters also requires more research. The American Society of Anesthesiologists (ASA) guidelines currently recommend allowing clear liquids up to 2 hours prior to elective procedures (Hausel et al., 2001). The majority of our survey respondents reported giving CCDs the morning of surgery, with half of these also administering it the night before.

## Conclusion

In summary, our study found that both simple sugar and complex sugar CCDs are commonly used within colorectal ERAS pathways. Surprisingly, we found substantial use of these CCDs, especially simple sugar CCDs, even in patients with diabetes who take insulin. Additional studies are needed to inform the ERAS community whether this variability in practice is deleterious.

## Supplementary Information

**Additional file 1:**

## Data Availability

The datasets used are not available.

## Abbreviations

ASA: American Society of Anesthesiologists
ASER: American Society for Enhanced Recovery
CCD: Carbohydrate-containing drink
CHO: Carbohydrate
ERAS: Enhanced recovery after surgery
IRB: Institutional review board
