# Supplementary material for: A U.S. survey of pre-operative carbohydrate-containing beverage use in colorectal enhanced recovery after surgery (ERAS) programs
Source: Perioper Med (Lond). 2021 May 28;10:19. doi: 10.1186/s13741-021-00187-3 (PMC8161920; doi:10.1186/s13741-021-00187-3)
Supplement: Supplementary file 1 — Additional file 1: [file 13741_2021_187_MOESM1_ESM.docx]

**Enhanced Recovery After Surgery (ERAS) Population**

1. Please check **each** service line for which you have an active Adult ERAS program: *(Select all that apply)*

□ Colorectal □ Cardiac □ Obstetric □ Spine □ ENT

□ Surgical Oncology □ Thoracic □ Gynecology □ Craniotomy

□ Orthopedic □ Bariatric □ Vascular □ Urology □ Plastic □ Other: ______________________ □ None (If you selected NONE please skip to Question 6)

1. Which of your Adult ERAS services utilize a pre-operative **carbohydrate (CHO)-containing** beverage?

*(Select all that apply)*

□ Colorectal □ Cardiac □ Obstetric □ Spine □ ENT

□ Surgical Oncology □ Thoracic □ Gynecology □ Craniotomy

□ Orthopedic □ Bariatric □ Vascular □ Urology □ Plastic

□ Other: ______________________ □ None (If you selected NONE please skip to Question 6)

1. For your Adult Colorectal ERAS program, which patients are asked to drink a **carbohydrate-containing** beverage? *(Select all that apply)*

| a) □ Non-Diabetics | b) □ Non-Insulin Dependent Diabetics | c) □ Insulin Dependent Diabetics |
| --- | --- | --- |
| *Please identify which CHO beverage is used most frequently: (Select one)* | *Please identify which CHO beverage is used most frequently: (Select one)* | *Please identify which CHO beverage is used most frequently: (Select one)* |
| □ Apple Juice  □ ClearFast™  □ Enhanced Medical Nutrition PREcovery™  □ Ensure Pre-Surgery™  □ Gatorade™  □ Gatorade Prime™  □ Glycemic Endothelial Drink (G.E.D)™  □ Lemonade  □ SurgiStrong RecoverAid™  □ Other: | □ Apple Juice  □ ClearFast™  □ Enhanced Medical Nutrition PREcovery™  □ Ensure Pre-Surgery™  □ Gatorade™  □ Gatorade Prime™  □ Glycemic Endothelial Drink (G.E.D)™  □ Lemonade  □ SurgiStrong RecoverAid™  □ Other: | □ Apple Juice  □ ClearFast™  □ Enhanced Medical Nutrition PREcovery™  □ Ensure Pre-Surgery™  □ Gatorade™  □ Gatorade Prime™  □ Glycemic Endothelial Drink (G.E.D)™  □ Lemonade  □ SurgiStrong RecoverAid™  □ Other: |

1. For your Adult Colorectal ERAS Program, when do patients drink the preoperative carbohydrate-containing beverage?

*(Select one)*

□ night before surgery only

□ morning of surgery only

□ night before surgery and morning of surgery

□ Other: _______________________________

1. Do most of your other Adult ERAS pathways use the same carbohydrate-containing drink as your Colorectal patients?

□ Yes

□ No, we use ___________________________

**Hospital Demographics**

1. With which do you identify: *(Select one)*

□ Anesthesiologist □ Surgeon □ Physician Assistant □ Nurse Practitioner

□ Nurse □ ERAS Coordinator □ Pharmacist

□ Other: _______________________________

1. How many Operating Rooms does the main hospital in which you work have? *(Select one)*

□ 1-10 ORs □ 11-20 ORs □ 21-30 ORs

□ 31-40 ORs □ 41-50 ORs □ >50 ORs

1. Does your main hospital have residents? *(Select one)*

□ None □ Anesthesia Only □ Surgery Only □ Anesthesia and Surgery

1. Name of the main hospital for which your answers reflect. This is for tracking purposes only and will not be disclosed in any publication or presentation.

_______________________________________
